# Construction of Dual-Shell Mo_2_C/C Microsphere towards Efficient Electromagnetic Wave Absorption

**DOI:** 10.3390/ijms232314502

**Published:** 2022-11-22

**Authors:** Xuesong Deng, Yahui Wang, Lifang Ma, Zhigang Li, Zongsheng Chen, Xiangyin Lv, Yajing Chang, Yi Liu, Jiaming Shi

**Affiliations:** 1 Anhui Provincial Laboratory of Advanced Laser Technology, National University of Defense Technology, Hefei 230037, China; 2 Science and Technology on Near-Surface Detection Laboratory, Wuxi 214000, China; 3 High Overload Ammunition Guidance and Control and Information Perception Laboratory, PLA Army Academy of Artillery and Air Defense, Hefei 230037, China

**Keywords:** Mo_2_C/C composites, dielectric loss, microwave absorption, dual-shell structure, polarization loss

## Abstract

Carbon-based carbides have attracted tremendous attention for electromagnetic energy attenuation due to their adjustable dielectric properties, oxidation resistance, and good chemical stability. Herein, we reasonably regulate the growth of dopamine hydrochloride on the surface of the Mo-glycerate (Mo-GL) microsphere and then transform the resultant Mo-polydopamine (Mo-PD) microsphere into a dual-shell Mo_2_C/C (DS-Mo_2_C/C) microsphere in a high-temperature pyrolysis process under an inert atmosphere. It is found that the pyrolysis temperature plays an important role in the graphitization degree of the carbon matrix and internal architecture. The fabrication of a dual-shell structure can be propitious to the optimization of impedance matching, and the introduction of Mo_2_C nanoparticles also prompts the accumulation of polarization loss. When the pyrolysis temperature reaches 800 °C, the optimized composite of DS-Mo_2_C/C-800 exhibits good EM absorption performance in the frequency range of 2.0–18.0 GHz. DS-Mo_2_C/C-800′s qualified bandwidth can reach 4.4 GHz at a matching thickness of 1.5 mm, and the integrated qualified bandwidth (QBW) even exceeds 14.5 GHz with a thickness range of 1.5–5.0 mm. The positive effects of the dual-shell structure and Mo_2_C nanoparticles on EM energy attenuation may render the DS-Mo_2_C/C microsphere as a promising candidate for lightweight and broad bandwidth EM absorption materials in the future.

## 1. Introduction

The application of advanced electronic instruments, especially high-power electromagnetic equipment, causes excessive electromagnetic (EM) pollution, which not only interferes with industrial production but may also endanger the health of biological systems [1,2,3,4]. EM absorption is the desired approach to alleviate EM pollution through energy conversion. During the past few decades, numerous investigations have been devoted to achieving highly efficient EM absorption properties through the construction of composites, such as magnetic metal composites, conducting polymer composites, and carbon-based composites [5,6,7,8]. Among the various composites, carbon-based magnetic composites have dominated the development of EM absorption materials. The characteristics of low density, chemical stability, and diversification in the carbon matrix bring tailorable performance, and the optimized combination of magnetic loss and dielectric loss is also conducive to EM energy attenuation [9,10,11,12]. However, these composites still possess a high density and inferior anticorrosion drawbacks in some cases. Meanwhile, dielectric loss always dominates microwave absorption in carbon-based magnetic composites because the existence of the carbon matrix significantly weakens the magnetic interaction between magnetic particles [13,14]. Inspired by this fact, it is proposed to replace magnetic components with other dielectric components in a carbon matrix, and a couple of studies have validated that binary dielectric composites can hoist EM absorption performance [15,16,17].

Recently, as a kind of potential dielectric material, carbon-based carbide composites have attracted tremendous attention for EM energy attenuation due to their adjustable dielectric properties, oxidation resistance, and good chemical stability [18,19,20]. Remarkably, the appearance of Mo_2_C promotes the potential application of carbon-based carbides in EM wave absorption fields [18,21,22]. Compared with other carbides, e.g., SiC or Ti_3_C_2_ MXenes, the relatively low pyrolysis temperature of Mo_2_C nanoparticles is beneficial for restraining crystal growth and facilitating homogeneous distribution [23,24]. In general, homogeneously dispersed small-size Mo_2_C nanoparticles are highly desirable for designing heterostructured carbon-based carbide composites because they are quite conducive to contributing sufficient polarization loss for EM energy attenuation [25]. For example, Dai et al. synthesized porous-carbon-based Mo_2_C composites through a MOFs-derived process. It was shown that affluent polarization loss and remarkable conductive loss contribute to improving microwave absorption performance [18].

Moreover, our group fabricated polyhedral morphology Mo_2_C/C composites through Mo-substituted ZIF-8. It was found that the uniform distribution of Mo_2_C nanoparticles in the carbon matrix can provide affluent interfacial polarizations to improve the EM absorption properties effectively. In addition, the as-prepared Mo_2_C/C composites have been proven to have good high-temperature stability and corrosion resistance [21]. Subsequently, we also regulated the structure of Mo_2_C/C and obtained core-shell Mo_2_C@C composites, which indicated the good microwave absorption performance of Mo_2_C@C ascribed to the conductivity loss and interfacial polarization loss [22]. Nevertheless, these Mo_2_C/C composites always imposed restrictions on structure design, mainly concentrated in a simple recombination or single core-shell structure, resulting in relatively narrow qualified response bandwidth and poor impedance matching. Recently, some researchers have demonstrated that dual-shell nanostructure, as a kind of intriguing nanostructure, has played a unique advantage in microwave fields by virtue of the pore structure, relatively low density, and affluent interfacial polarization [26,27,28,29]. It has been found that the dual-shell structure is propitious to the optimization of impedance matching because the encapsulated air between dual shells can act as a mediator to prompt intrinsic impedance as close as possible to that of outside air [29]. Moreover, the double-cavity structure strengthens reflection loss (RL) by extending the propagation path of the incident EM waves to promote their deflection and scattering [30,31]. Therefore, it will be a gratifying exploration to develop a rational route for introducing dual-shell architecture into Mo_2_C/C composites to realize a unique heterostructure EM system with optimized impedance matching and enhanced EM absorption performance.

Herein, we reasonably regulate the growth of dopamine hydrochloride on the surface of Mo-glycerate (Mo-GL) microspheres and then transform the resultant Mo-polydopamine (Mo-PD) microspheres into dual-shell architecture Mo_2_C/C (DS-Mo_2_C/C) microspheres in a high-temperature pyrolysis process under an inert atmosphere. The final Mo_2_C/C microspheres exhibit a dual-shell hollow structure, where Mo_2_C nanoparticles are decorated in both shells of the carbon matrix. The graphitization degree and microwave absorption properties of the DS-Mo_2_C/C microsphere can be well tailored through pyrolysis temperature. The optimized DS-Mo_2_C/C-800 (the corresponding pyrolysis temperature of 800 °C) satisfactorily possesses a gratifying response bandwidth of 4.8 GHz with a small applied thickness of 1.42 mm. The favorable EM absorption property of DS-Mo_2_C/C-800 not only benefits from its intrinsic loss, but its internal heterostructure can also improve auxiliary EM energy attenuation.

## 2. Results and Discussion

### 2.1. Material Characteristics

**Figure 1:**
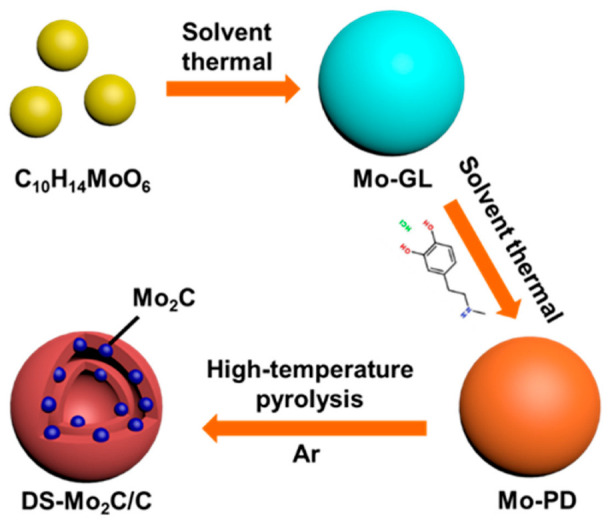
Schematic illustration of preparing DS-Mo_2_C/C microsphere.

**Figure 2:**
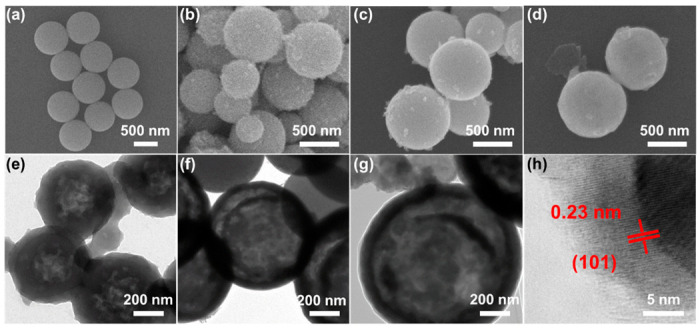
SEM image of Mo-PD (**a**); SEM images of DS-Mo_2_C/C microsphere at different pyrolysis temperatures: 700 °C (**b**), 800 °C (**c**), and 900 °C (**d**); TEM images of DS-Mo_2_C/C microsphere at different pyrolysis temperatures: 700 °C (**e**), 800 °C (**f**), and 900 °C (**g**); HRTEM image of 800 °C (**h**).

The strategy for synthesizing the DS-Mo_2_C/C microsphere is schematically depicted in Figure 1. Firstly, uniform Mo-GL microspheres with a diameter of ~700 nm are prepared through a moderate solvothermal process (Figure S2). Subsequently, ammonia solution is employed to initiate the polymerization of dopamine hydrochloride on the surface of Mo-GL to form the microsphere of dopamine hydrochloride contained inside Mo-GL, which was artificially named Mo-PD (Figure 2a). As shown in Figure S3, one can see that a slight dissolution inside the sphere accompanies the polymerization process. The obtained Mo-PD microspheres are subsequently pyrolyzed at the required temperatures within the Ar atmosphere. At the same time, the Mo-PD microsphere is converted into Mo_2_C and a carbon shell with core-shell by pyrolysis treatments.

Figure 2b–d show the low-magnification SEM images of the DS-Mo_2_C/C microsphere with different pyrolysis temperatures. It is clear that the resultant DS-Mo_2_C/C presents a very uniform spherical morphology with smooth surfaces and spherical sizes around 700 nm and commendably maintains the original profile of their precursors (Figure S2). Moreover, there is no apparent heterogeneous architecture around DS-Mo_2_C/C-700, DS-Mo_2_C/C-800, and DS-Mo_2_C/C-900, indicating the formation of Mo_2_C nanoparticles will preferentially occur inside individual microspheres. TEM measurement was employed to identify the internal structure of the DS-Mo_2_C/C microsphere. The DS-Mo_2_C/C-700, DS-Mo_2_C/C-800, and DS-Mo_2_C/C-900 microspheres all display recognizable hollow structures, as shown in Figure 2e–g. Although they inherit the spherical morphology of the Mo-PD microsphere, the internal structure differs from the precursor (Figure S3). DS-Mo_2_C/C-700 presents a misty stratification cavity inside the microsphere, and there are also some tiny black blocks inside the carbon shell (Figure 2e). As the pyrolysis temperature reaches 800 °C, the sample retains a hollow structure but exhibits an apparent dual shell. Furthermore, there are fewer small black blocks inside the double carbon shell, and the cavity grows (Figure 2f). This structure may stimulate the reinforcement of multiple reflections and scatterings for incident EM waves, and the encapsulated air between dual shells can also optimize impedance matching. DS-Mo_2_C/C-900 remains spherical morphology and dual shell, but the inner shell appears to fracture (Figure 2g). In detail, the lattice fringes (0.23 nm) in Figure 2h can be affiliated to the (101) plane of hexagonal Mo_2_C [31,32].

**Figure 3:**
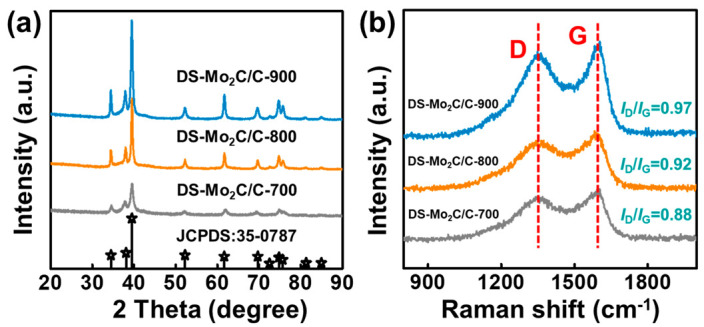
XRD patterns (**a**), Raman spectra (**b**) of DS-Mo_2_C/C.

Wide-angle XRD primarily studies the crystalline structures of DS-Mo_2_C/C-700, DS-Mo_2_C/C-800, and DS-Mo_2_C/C-900. The DS-Mo_2_C/C-700, DS-Mo_2_C/C-800, and DS-Mo_2_C/C-900 display different characteristic diffraction peaks at 34.4°, 38°, 39.4°, 52.1°, 61.5°, 69.6°, and 75°, as shown in Figure 3a, respectively [33,34], which correspond to the (100), (002), (101), (102), (110), and (103) planes of a hexagonal Mo_2_C (JCPDS 35-0787) [35]. In addition to these characteristic diffraction peaks of Mo_2_C, there are no other additional diffraction peaks assigned to molybdenum-containing compounds, implying a pure Mo_2_C phase can be well fabricated through this approach. The above results are consistent with the HRTEM image’s results, indicating the Mo_2_C crystals can be well obtained during the pyrolysis process. It can be seen that the relative intensities and sharpness of these diffraction peaks are dependent on the pyrolysis temperature, which indicates that the temperature variation may influence the growth of the Mo_2_C nanoparticle [36]. Interestingly, the half-peak width of Mo_2_C diffraction peaks becomes shrunken with the increase in temperature. As we all know, this variation also indicates the distinction in Mo_2_C particle size [33]. According to Scherrer’s formula [37],

(1) $$\bar{D}=\frac{0.94\lambda }{\beta \cos\theta }$$

where $\bar{D}$ and *λ* represent average crystalline size and radiation wavelength (0.154 nm herein), respectively; *β* is the half-peak width of the designated diffraction peak, and *θ* is the Bragg angle. It can be calculated that the sizes of Mo_2_C nanoparticles deduced from the most substantial diffraction peak of (101) plane of DS-Mo_2_C/C-700, DS-Mo_2_C/C-800, and DS-Mo_2_C/C-900 are 15.2, 20.5, and 25.3 nm, respectively because the nanocrystalline materials tended to aggregate at high temperatures and form united flakes with larger sizes [38].

The graphitization degree of carbon-based materials plays an essential role in EM absorption performance [39]. Raman spectra are necessary to discern the graphitization degree of DS-Mo_2_C/C-700, DS-Mo_2_C/C-800, and DS-Mo_2_C/C-900 (Figure 3b) [40]. One can see that the two distinguishable peaks at ~1350 and 1590 cm^−1^ correspond to the D and G bands of carbon [29,41]. Generally, the D band is assigned to the sp^3^ defect or disordered graphitic carbon, and the G band corresponds to the sp^2^-hybridized graphitic carbon layer [42,43]. The relative intensity ratio of the D band and G band (*I*_D_/*I*_G_) is employed to represent the graphitization of carbon-based materials. According to the reported literature [21], when tiny nanocrystalline graphite appears in the translation of amorphous carbon to graphite carbon, the *I*_D_/*I*_G_ values increase, which is because of the decrease in the bond angle and bond bending in disordered carbon atoms, and the correspondingly vibrational density of states is tighter. As shown in Figure 3b, the *I*_D_/*I*_G_ values of DS-Mo_2_C/C-700, DS-Mo_2_C/C-800, and DS-Mo_2_C/C-900 increase from 0.88 to 0.97, which implies a higher temperature will stimulate the improvement in their relative graphitization degree [43].

### 2.2. Electromagnetic Parameters and Performance Analysis

**Figure 4:**
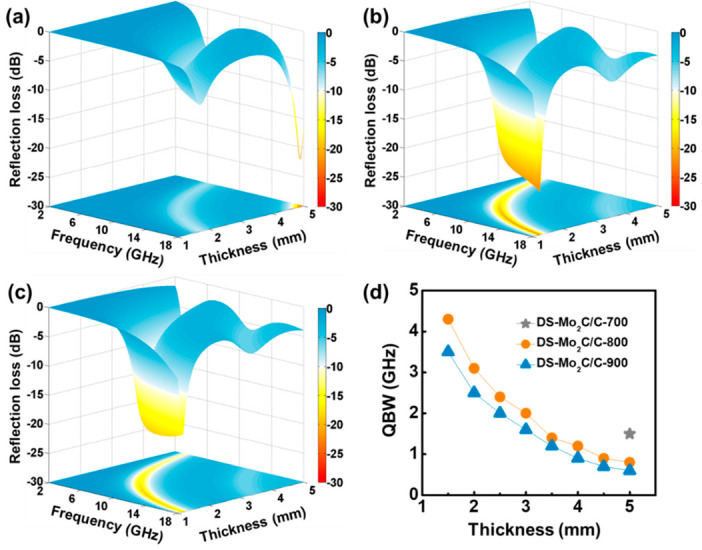
Three-dimensional reflection loss maps of DS-Mo_2_C/C-700 (**a**), DS-Mo_2_C/C-800 (**b**), and DS-Mo_2_C/C-900 (**c**); The QBW at the specific thickness of DS-Mo_2_C/C-700, DS-Mo_2_C/C-800, and DS-Mo_2_C/C-900 (**d**).

The relative complex permittivity (*ε*_r_) and the relative complex permeability (*μ*_r_) are two important parameters for calculating *RL* values and dominating the EM absorption performance of the as-prepared DS-Mo_2_C/C microsphere. According to the theory of transmission lines, the *RL* values of DS-Mo_2_C/C are calculated through the experimental permittivity and permeability with the following equations [15,18],

(2) $$RL\left(\mathrm{dB}\right)=20\log\left|\frac{z_{\mathrm{in}}-1}{z_{\mathrm{in}}+1}\right|$$

(3) $$Z_{\mathrm{in}}=\sqrt{\frac{\mu_{r}}{\varepsilon_{r}}}\tanh\left[j\left(\frac{2\pi }{c}\right)fd\sqrt{\mu_{r}\varepsilon_{r}}\right]$$

where *c* is the EM waves’ velocity in free space, and *f* and *d* represent frequency and artificially given absorber thickness, respectively [44]. Figure 4a–c show the three-dimensional *RL* maps of DS-Mo_2_C/C-700, DS-Mo_2_C/C-800, and DS-Mo_2_C/C-900 in the frequency of 2.0–18.0 GHz. When the pyrolysis temperature is 700 °C, the obtained DS-Mo_2_C/C-700 exhibits inferior EM attenuation ability. Although its minimum *RL* value reaches −22.0 dB at 17.7 GHz, the corresponding thickness is 5.0 mm, and the minimum *RL* value is less than −10 dB at 5 mm thickness only. The *RL* < −10 dB always signifies an acceptable EM energy attenuation level of >90% [14,45]. With the increase in temperature, DS-Mo_2_C/C-800 and DS-Mo_2_C/C-900 display enhanced EM absorption properties. The minimum *RL* value of DS-Mo_2_C/C-800 is −21.0 dB at 14.2 GHz with a thin thickness of 1.5 mm (Figure 4b). When the temperature reaches 900 °C, the minimum *RL* value cannot even reach −20.0 dB with a thickness of 1.0–5.0 mm. The minimum *RL* value is just −19.0 dB at 13.4 GHz with the thickness of 1.5 mm (Figure 4c). Obviously, the minimum *RL* values of DS-Mo_2_C/C-800 and DS-Mo_2_C/C-900 are inferior to that of DS-Mo_2_C/C-700. However, the corresponding thickness of DS-Mo_2_C/C-700 is 5.0 mm, which is not conducive to practical application. In addition to the minimum *RL* value, the frequency ranges with *RL* values less than −10.0 dB, i.e., the qualified bandwidth (QBW), are also a more critical index to characterize EM absorption performance. As expected, DS-Mo_2_C/C-800 has a superior QBW compared to DS-Mo_2_C/C-700 and DS-Mo_2_C/C-900 at specific thicknesses (Figure 4d). The optimized QBW of DS-Mo_2_C/C-800 can reach 4.4 GHz with a thickness of 1.5 mm. When the thicknesses are artificially restricted at the range of 1.0–5.0 mm, the total QBW corresponding to these thicknesses is always defined as integrated QBW [8,21,42]. The projection area of the three-dimensional *RL* map in the *x* and *y* planes, that is, the projection of the bottom of Figure 4a–c, can intuitively give the integrated QBW. The integrated QBW of DS-Mo_2_C/C-800 even exceeds 14.5 GHz, which almost contains the S, C, X, and Kµ bands. As shown in Figure S4, the meritorious integrated QBW of DS-Mo_2_C/C-800 exhibits as being more or less superior to some carbon-based EM absorption materials [15,46,47,48,49,50,51,52,53,54,55].

**Figure 5:**
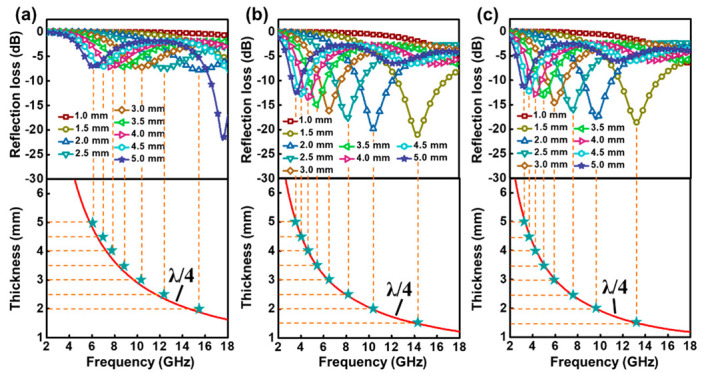
The relationship between the minimum *RL* values with different thicknesses of DS-Mo_2_C/C-700 (**a**), DS-Mo_2_C/C-800 (**b**), and DS-Mo_2_C/C-900 (**c**).

It is interesting that in the frequency region of 2.0–18.0 GHz, the minimum *RL* value gradually shifts from high frequency to low frequency with the increase in coating thickness (Figure 5a–c), which is related to the quarter-wavelength (*nλ*/4) matching model [49]. It means that when EM waves experience an absorber supported by a plate metal, there are two situations: one is that the surface of the air absorber reflects some EM waves, and the other is that the surface of the plate metal reflects slight EM waves. In some cases, the incident EM waves and the reflected waves can form 180° at the surface of the air absorber and offset each other. Based on the *λ*/4 matching model, the simulations of the matching thickness (*t*_m_) and corresponding frequency of the absorption peak (*f*_m_) conform to the following equation [50,56],

(4) $$t_{m}=\frac{n}{4}\lambda_{m}=\frac{nc}{4f_{m}\sqrt{\left|\varepsilon_{r}\mu_{r}\right|}}\;\left(n=1,\;3,\;5\ldots \right)$$

where *λ*_m_ is the quarter-wavelength at *f*_m_, and *c* is the velocity of EM waves in free space. Figure 5a–c display the curves of *RL* values and *t*_m_ vs. *f*_m_ of DS-Mo_2_C/C-700, DS-Mo_2_C/C-800, and DS-Mo_2_C/C-900, respectively. The cyan pentacles are the minimum *RL* values at certain thicknesses derived from transmission line theory, and the rose curves represent the *t*_m_ calculated from a quarter-wavelength equation. Without a doubt, the cyan pentacles are whole along the rose curve, verifying the relationship between *t*_m_ and *f*_m_ for the EM absorption of DS-Mo_2_C/C-700, DS-Mo_2_C/C-800, and DS-Mo_2_C/C-900 composites, obeying the *nλ*/4 model.

**Figure 6:**
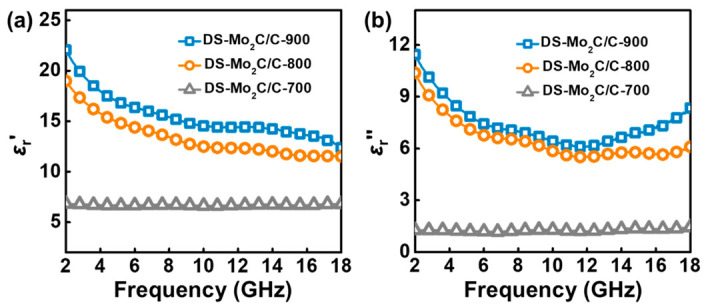
Real parts (**a**) and imaginary parts (**b**) of the complex permittivity of DS-Mo_2_C/C-700, DS-Mo_2_C/C-800, and DS-Mo_2_C/C-900.

EM parameters can provide an intuitive approach to revealing the difference in microwave absorption performance. The properties of EM absorption materials are highly associated with their *ε*_r_ and *μ*_r_, where the real parts of complex permittivity (*ε*_r_′) and complex permeability (*μ*_r_′) represent the storage capability of electric and magnetic energy, and the imaginary parts (*ε*_r_″ and *μ*_r_″) stand for the loss capability of electric and magnetic energy [53,57]. As expected, it can be found that all DS-Mo_2_C/C microspheres are unable to generate magnetic loss because of the absence of magnetic components, where the values of *μ*_r_′ and *μ*_r_″ are approximate constants and close to 1 and 0, respectively (Figure S5). Thus, relative complex permittivity will dominate as the foundation for attenuating EM energy. Figure 6a shows the different *ε*_r_′ values of DS-Mo_2_C/C-700, DS-Mo_2_C/C-800, and DS-Mo_2_C/C-900. Figure 6a,b show *ε*_r_′ and *ε*_r_″ values of DS-Mo_2_C/C-700, DS-Mo_2_C/C-800, and DS-Mo_2_C/C-900 in the frequency range 2.0–18.0 GHz, respectively. The *ε*_r_′ and *ε*_r_″ values of DS-Mo_2_C/C-700 are almost constant throughout the whole frequency range, especially where *ε*_r_″ values are below 3.0, which indicates the inadequate ability of EM energy attenuation. With the increase in temperature, both the *ε*_r_′ and *ε*_r_″ values of DS-Mo_2_C/C-800 and DS-Mo_2_C/C-900 exhibit noticeable enhancement and show the apparent behavior of frequency dependency. For example, the *ε*_r_′ values of DS-Mo_2_C/C-800 and DS-Mo_2_C/C-900 decreased from 18.98 to 11.52 and 22.06 to 12.38, respectively, and the corresponding *ε*_r_″ values declined from 10.38 to 6.14 and 11.45 to 8.39, respectively. It has to be pointed out that most carbon-based materials generally display a frequency dispersion behavior in the range of 2.0–18.0 GHz because of polarization hysteresis induced by the high-frequency alternating EM field [54,55,58,59,60,61,62]. However, the *ε*_r_″ values of DS-Mo_2_C/C-800 and DS-Mo_2_C/C-900 show abnormal dispersion behaviors in the range of 12.0–18.0 GHz and cut off at about 12.0 GHz, which mainly originates from interfacial polarization occurring at 12.0–18.0 GHz [18,63]. Dielectric loss specifically includes conductivity loss and polarization loss, where conductivity loss relates to conductibility and polarization loss usually originates from interfacial polarization and dipole orientation polarization [11,28]. To validate the contribution of conductance loss and polarization loss, we further introduce the nonlinear equation as follows [64,65],

(5) $$\varepsilon_{r}^{\prime \prime }=\varepsilon_{p}^{\prime \prime }+\varepsilon_{c}^{\prime \prime }=\left(\varepsilon_{s}-\varepsilon_{\infty }\right)\frac{2\pi f\sigma }{1+{\left(2\pi f\right)}^{2}\tau^{2}}+\frac{\sigma }{2\pi f\varepsilon_{0}}$$

where $\varepsilon_{p}^{\prime \prime }$ and $\varepsilon_{c}^{\prime \prime }$ are the parts of the contribution in polarization and conductance loss, respectively, $\varepsilon_{s}$ and $\varepsilon_{\infty }$ represent the static dielectric constant and dielectric constant at infinite frequency, respectively. Polarization loss can be verified through Debye theory, and the Cole-Cole semicircle can be formulated by the following [66,67],

(6) $${\left(\varepsilon_{r}^{\prime }-\frac{\varepsilon_{s}+\varepsilon_{\infty }}{2}\right)}^{2}+\varepsilon_{r}^{\prime \prime 2}={\left(\frac{\varepsilon_{s}-\varepsilon_{\infty }}{2}\right)}^{2}$$

The $\varepsilon_{r}^{\prime \prime }\;vs.\;\varepsilon_{r}^{\prime }$ curves of DS-Mo_2_C/C-700, DS-Mo_2_C/C-800, and DS-Mo_2_C/C-900 are plotted in Figure S6a–c according to Equation (6). One can see that three samples give distinct semicircles and also exhibit a different-slope straight line at the region of high $\varepsilon_{r}^{\prime }$ values. According to Equation (5), when the contribution of conductance loss to dielectric loss is insufficient, the Cole-Cole semicircle will dominate the whole curve with severe distortions. As shown in Figure S6a, DS-Mo_2_C/C-700 possesses several visible semicircles, which may come from multiple relaxation processes, implying conductance loss will only give a faintly auxiliary contribution to dielectric loss. With the increase in temperature, DS-Mo_2_C/C-800 and DS-Mo_2_C/C-900 display straight lines at the end of the Cole-Cole semicircle, which indicates conductance loss dominating the contribution for dielectric loss due to the improvement in graphitization degree. These results show that the appreciative dielectric loss of DS-Mo_2_C/C mainly contributes to the synergetic effect of conductance loss and polarization loss.

Moreover, as well as dielectric loss, we further introduce the attenuation constant (α) to describe the overall EM attenuation capability as the following equation [68],

(7) $$\alpha =\frac{\sqrt{2}\pi f}{c}\sqrt{\left(\mu_{r}^{\prime \prime }\varepsilon_{r}^{\prime \prime }-\mu_{r}^{\prime }\varepsilon_{r}^{\prime }\right)+\sqrt{{\left(\mu_{r}^{\prime \prime }\varepsilon_{r}^{\prime \prime }-\mu_{r}^{\prime }\varepsilon_{r}^{\prime }\right)}^{2}+{\left(\mu_{r}^{\prime }\varepsilon_{r}^{\prime \prime }+\mu_{r}^{\prime \prime }\varepsilon_{r}^{\prime }\right)}^{2}}}$$

**Figure 7:**
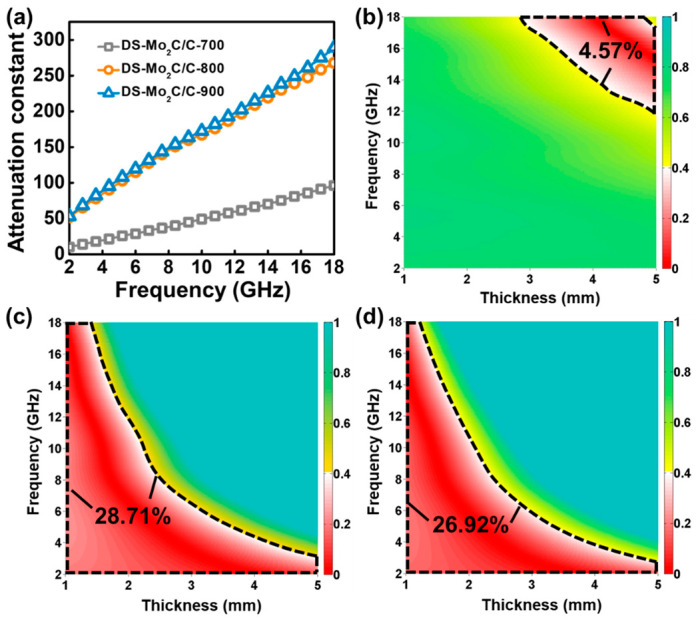
The attenuation constant for DS-Mo_2_C/C (**a**); calculated delta value maps of DS-Mo_2_C/C-700 (**b**), DS-Mo_2_C/C-800 (**c**), and DS-Mo_2_C/C-900 (**d**).

Figure 7a shows the values of *α* vs. frequency for DS-Mo_2_C/C-700, DS-Mo_2_C/C-800, and DS-Mo_2_C/C-900. All samples present a monotonous increase in the *α* value in the frequency scope (2.0–18.0 GHz), implying that incident EM waves may be easier to weaken at a high-frequency range. For example, the *α* values of DS-Mo_2_C/C-700 increased from 10.1 to 96.3. It is worth noting that DS-Mo_2_C/C-800 and DS-Mo_2_C/C-900 possess similar *α* values, where their *α* values increased from 51.8 and 53.1 to 267.8 and 289.1, respectively. These *α* values’ variation obeys the order of $\varepsilon_{r}^{\prime \prime }$ and $\tan\delta_{e}$ values (DS-Mo_2_C/C-700<DS-Mo_2_C/C-800<DS-Mo_2_C/C-900), revealing that dielectric loss dominates the EM energy attenuation. Although DS-Mo_2_C/C-900 has meritorious dielectric loss capability, its EM absorption performance is inferior to that of DS-Mo_2_C/C-800. The precondition of good EM absorption performance is that the surfaces of EM absorption materials and free space have good impedance matching, that is, almost zero reflection of incident EM waves on the surface of EM absorption materials and the guarantee that most EM waves can be consumed [69]. The delta function (|Δ|) is always employed to describe the characteristic of impedance between free space and EM absorption materials as the following equations [70,71,72],

(8) $$\left|\Delta \right|=\left|\sinh^{2}\left(Kfd\right)-M\right|$$

(9) $$K=\frac{4\pi \sqrt{\mu_{r}^{\prime }\varepsilon_{r}^{\prime }}\sin\frac{\delta_{e}+\delta_{m}}{2}}{c\cos\delta_{e}\cos\delta_{m}}$$

(10) $$M=\frac{4\mu_{r}^{\prime }\cos\delta_{e}\varepsilon_{r}^{\prime }\cos\delta_{m}}{{\left(\mu_{r}^{\prime }\cos\delta_{e}-\varepsilon_{r}^{\prime }\cos\delta_{m}\right)}^{2}+{\left[\tan\left(\frac{\delta_{m}}{2}-\frac{\delta_{e}}{2}\right)\right]}^{2}{\left(\mu_{r}^{\prime }\cos\delta_{e}+\varepsilon_{r}^{\prime }\cos\delta_{m}\right)}^{2}}$$

Figure 7b–d show the two-dimension |Δ| maps of DS-Mo_2_C/C-700, DS-Mo_2_C/C-800, and DS-Mo_2_C/C-900 with thickness and frequency as the *x* and *y* axes, and it continually defines |Δ| ≤ 0.4 as desirable impedance matching [21,73]. Observably, DS-Mo_2_C/C-700 gives an inferior matching region with an effective coverage ratio of ~4.75%, and, meanwhile, the insufficient dielectric loss capability cannot bring remarkable EM absorption properties (Figure 4a and Figure 6). When the temperature reaches 700 °C, DS-Mo_2_C/C-800 exhibits superior impedance matching with the matching region, about 28.71%. Unfortunately, DS-Mo_2_C/C-900 presents a decreasing impedance matching characteristic, and its effective matching region is approximately 26.92%. Although DS-Mo_2_C/C-900 has the highest *α* value (Figure 7a), the impedance matching inferior to that of DS-Mo_2_C/C-800 makes most incident EM waves reflect into the air at the upper interface of Mo_2_C/C-900 (air absorber interface), leading to the poor EM absorption performance. Thus, the remarkable EM absorption property of DS-Mo_2_C/C-800 mainly benefits from desirable impedance matching and proper attenuation capability.

**Figure 8:**
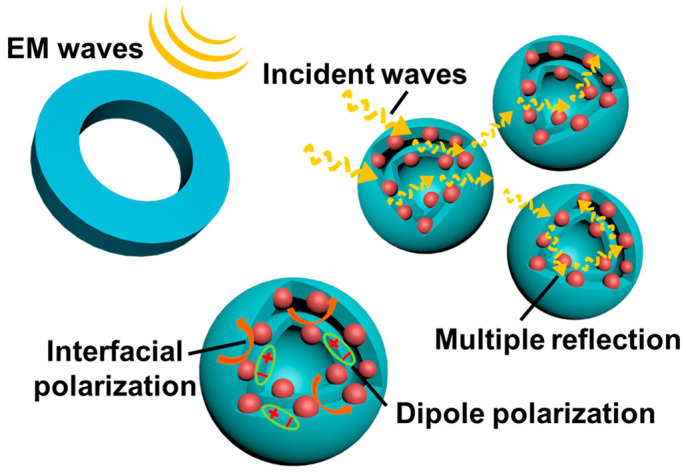
The schematic illustration of the physical model for microwave absorption mechanisms in DS-Mo_2_C/C nanosphere.

A physical model diagram is a promising approach to ascertaining the EM absorption mechanism of the DS-Mo_2_C/C nanosphere. As shown in Figure 8, most incident waves will penetrate the surface of the absorber containing the DS-Mo_2_C/C nanosphere and undergo a series of attenuations. One can see that the propagation route of incident EM waves can be effectively extended through the dual-shell structure among neighboring microspheres. This complex propagation route brings multiple reflections and the diffuse scattering of incident EM waves, resulting in repeated EM energy attenuation. Introducing Mo_2_C nanoparticles also provides sufficient heterointerface and dipole centers, generating abundant interfacial polarization and gratifying dipole polarization to consume EM energy.

## 3. Materials and Methods

### 3.1. Synthesis of Mo-GL Spheres

According to the previous report [31], Mo-GL microspheres were prepared by magnetic stirring and subsequent solvothermal reaction by dissolving 245 mg of molybdenum acetoacetate and 16 mL of G.L. in a mixed solution consisting of 15 mL of deionized water and 65 mL of isopropanol, which was sonicated until dissolved to a transparent solution. Subsequently, the transparent solution was transferred into a 150 mL Teflon-lined autoclave and heated at 160 °C for 5 h in an oven. The obtained powder was collected by centrifugation, washed with ethanol several times, and dried at room temperature.

### 3.2. Synthesis of Mo-GL Spheres

In total, 20 mg of Mo-GL and 20 mg of dopamine hydrochloride were dispersed in a mixture solution with 8 mL of absolute alcohol and 6 mL of deionized water under sonication. Then 20 μL of ammonia solution was quickly decanted into a dispersion solution with magnetic stirring. The dispersion solution was transferred into a 50 mL Teflon-lined autoclave and heated at 140 °C for 2 h. Finally, the as-obtained Mo-PD composites were pyrolyzed in a horizontally tubular furnace under an Ar atmosphere at the required temperature for 3 h with a 2 °C/min heating rate. The final composites were named DS-Mo_2_C/C-700, DS-Mo_2_C/C-800, and DS-Mo_2_C/C-900 according to the pyrolysis temperature at 700, 800, and 900 °C, respectively.

### 3.3. Characterization

Powder X-ray diffraction (XRD) data were recorded on a Rigaku D/MAXRC X-ray diffractometer with a Cu Ka radiation source (40.0 kV, 40.0 mA). Raman spectra were measured on a confocal Raman spectroscopic system (Renishaw, In Via, the UK) using a 532 nm laser. Scanning electron microscopy (SEM) images were obtained on a Quanta 200S (FEI), and the samples were mounted on aluminum studs by using adhesive graphite tape and sputter coated with gold before analysis. Transmission electron microscopy (TEM) images were obtained on a Tecnai F20 operating at an accelerating voltage of 200 kV. An Agilent PNA-N5244A vector network analyzer (Agilent, the USA) was applied to determine the relative permeability and permittivity in the frequency range of 2.0–18.0 GHz for the calculation of RL. The measurement process of EM absorption properties can be divided into two steps: (1) before the measurement, 40wt% of the obtained composites and 60wt% of molten paraffin wax were adequately grinded for about 30 min to obtain a uniform mixture. Then the mixture was collected in an artificial mold for pressing into a ring with an outer diameter of 7.0 mm, an inner diameter of 3.0 mm, and a thickness of 2.0 mm (Figure S1a). (2) We provided the instrument settings of the Ceyear AV3672C as a demonstration, and its parameters and procedure are the same as those of Agilent PNA-N5244A. Measurements setup consists of a VNA working up to 43.5 GHz, a coaxial calibration kit in the 2–18 GHz, sample holder hosting the materials under test (Figure S1b). In the process of measurement, we put the concentric annulus (with a thickness of 2 mm) into the sample holder, and the experimental result is translated into permittivity and permeability with the number of frequency steps 201 at the frequency of 2–18 GHz.

## 4. Conclusions

Dual-shell Mo_2_C/C microspheres are rationally fabricated by regulating the growth of dopamine hydrochloride on the surface of the Mo-glycerate (Mo-GL) microsphere and then undergo a high-temperature pyrolysis process. Introducing Mo_2_C nanoparticles produces a positive impact on the dielectric loss of these composites, where they accumulate considerable interfacial polarization. More importantly, the dual-shell structure optimizes impedance matching by prompting intrinsic impedance as close as possible to that of the outside air. The good balance between dielectric loss and impedance matching can produce appreciative EM absorption properties by tailoring the pyrolysis temperature. The EM attenuation ability of as-resultant DS-Mo_2_C/C-800 is superior to those of many reported carbon-based composites, and gives not only a broad QBW with 4.4 GHz at the thickness of 1.5 mm but also possesses a gratifying integrated QBW of 14.6 GHz in the thickness range of 1.5–5.0 mm. We believe these results might stimulate the design and preparation of highly efficient carbon-based EM absorption materials with specific structures.

## Data Availability

The authors declare that all data supporting the findings are available within the paper or are available from the authors upon request.

## Supplementary Materials

The following supporting information can be downloaded at: https://www.mdpi.com/article/10.3390/ijms232314502/s1, Figure S1: The pictures of the coaxial calibration kit and sample (a), and measurement process of Ceyear AV3672C (b); Figure S2: SEM image of Mo-GL microsphere; Figure S3: TEM image of Mo-PD microsphere; Figure S4: The integrated QBW at the range of 2.0–18.0 GHz of DS-Mo_2_C/C-800 and other carbon-based EM absorption materials. Figure S5: Real parts (a) and imaginary parts (b) of the complex permeability of DS-Mo_2_C/C-700, DS-Mo_2_C/C-800, and DS-Mo_2_C/C-900; Figure S6: The Cole-Cole semicircle of DS-Mo_2_C/C-700 (a), DS-Mo_2_C/C-800 (b), and DS-Mo_2_C/C-900 (c).

## References

[1] Chen Y., Potschke P., Pionteck J., Voit B., Qi H. (2020). Multifunctional Cellulose/rGO/Fe_3_O_4_ Composite Aerogels for Electromagnetic Interference Shielding. ACS Appl. Mater. Interfaces.

[2] Liu P., Gao S., Wang Y., Huang Y., Wang Y., Luo J. (2019). Core-Shell CoNi@Graphitic Carbon Decorated on B,N-Codoped Hollow Carbon Polyhedrons toward Lightweight and High-Efficiency Microwave Attenuation. ACS Appl. Mater. Interfaces.

[3] Wang X.X., Cao W.Q., Cao M.S., Yuan J. (2020). Assembling Nano-Microarchitecture for Electromagnetic Absorbers and Smart Devices. Adv. Mater..

[4] Wang J., Liu L., Jiao S., Ma K., Lv J., Yang J. (2020). Hierarchical Carbon Fiber@MXene@MoS_2_ Core-sheath Synergistic Microstructure for Tunable and Efficient Microwave Absorption. Adv. Funct. Mater..

[5] Liu Q., Cao Q., Bi H., Liang C., Yuan K., She W., Yang Y., Che R. (2016). CoNi@SiO_2_@TiO_2_ and CoNi@Air@TiO_2_ Microspheres with Strong Wideband Microwave Absorption. Adv. Mater..

[6] Lv J., Liang X., Ji G., Quan B., Liu W., Du Y. (2018). Structural and Carbonized Design of 1D FeNi/C Nanofibers with Conductive Network to Optimize Electromagnetic Parameters and Absorption Abilities. ACS Sustain. Chem. Eng..

[7] Li Z., Han X., Ma Y., Liu D., Wang Y., Xu P., Li C., Du Y. (2018). MOFs-Derived Hollow Co/C Microspheres with Enhanced Microwave Absorption Performance. ACS Sustain. Chem. Eng..

[8] Tian C., Du Y., Xu P., Qiang R., Wang Y., Ding D., Xue J., Ma J., Zhao H., Han X. (2015). Constructing Uniform Core-Shell PPy@PANI Composites with Tunable Shell Thickness toward Enhancement in Microwave Absorption. ACS Appl. Mater. Interfaces.

[9] Wang K., Chen Y., Tian R., Li H., Zhou Y., Duan H., Liu H. (2018). Porous Co-C Core-Shell Nanocomposites Derived from Co-MOF-74 with Enhanced Electromagnetic Wave Absorption Performance. ACS Appl. Mater. Interfaces.

[10] Wu T., Liu Y., Zeng X., Cui T., Zhao Y., Li Y., Tong G. (2016). Facile Hydrothermal Synthesis of Fe_3_O_4_/C Core-Shell Nanorings for Efficient Low-Frequency Microwave Absorption. ACS Appl. Mater. Interfaces.

[11] Zhao H., Wang F., Cui L., Xu X., Han X., Du Y. (2021). Composition Optimization and Microstructure Design in MOFs-Derived Magnetic Carbon-Based Microwave Absorbers: A Review. Nano-Micro Lett..

[12] Guo T., Huang B., Li C., Lou Y., Tang X.-Z., Huang X., Yue J. (2021). Magnetic sputtering of FeNi/C bilayer film on SiC fibers for effective microwave absorption in the low-frequency region. Ceram. Int..

[13] Liu Q., Liu X., Feng H., Shui H., Yu R. (2017). Metal organic framework-derived Fe/carbon porous composite with low Fe content for lightweight and highly efficient electromagnetic wave absorber. Chem. Eng. J..

[14] Lu Y., Wang Y., Li H., Lin Y., Jiang Z., Xie Z., Kuang Q., Zheng L. (2015). MOF-Derived Porous Co/C Nanocomposites with Excellent Electromagnetic Wave Absorption Properties. ACS Appl. Mater. Interfaces.

[15] Tian X., Meng F., Meng F., Chen X., Guo Y., Wang Y., Zhu W., Zhou Z. (2017). Synergistic Enhancement of Microwave Absorption Using Hybridized Polyaniline@helical CNTs with Dual Chirality. ACS Appl. Mater. Interfaces.

[16] Chen H., Huang Z., Huang Y., Zhang Y., Ge Z., Qin B., Liu Z., Shi Q., Xiao P., Yang Y. (2017). Synergistically assembled MWCNT/graphene foam with highly efficient microwave absorption in both C and X bands. Carbon.

[17] Zhao B., Deng J., Liang L., Zuo C., Bai Z., Guo X., Zhang R. (2017). Lightweight porous Co_3_O_4_ and Co/CoO nanofibers with tunable impedance match and configuration-dependent microwave absorption properties. Cryst. Eng. Comm..

[18] Dai S., Cheng Y., Quan B., Liang X., Liu W., Yang Z., Ji G., Du Y. (2018). Porous-Carbon-Based Mo_2_C Nanocomposites as Excellent Microwave Absorber: A New Exploration. Nanoscale.

[19] Han M., Yin X., Li X., Anasori B., Zhang L., Cheng L., Gogotsi Y. (2017). Laminated and Two-Dimensional Carbon-Supported Microwave Absorbers Derived from MXenes. ACS Appl. Mater. Interfaces.

[20] Tong Y., He M., Zhou Y., Nie S., Zhong X., Fan L., Huang T., Liao Q., Wang Y. (2018). Three-Dimensional Hierarchical Architecture of the TiO_2_/Ti_3_C_2_T_x_/RGO Ternary Composite Aerogel for Enhanced Electromagnetic Wave Absorption. ACS Sustain. Chem. Eng..

[21] Wang Y., Li C., Han X., Liu D., Zhao H., Li Z., Xu P., Du Y. (2018). Ultrasmall Mo_2_C Nanoparticle-Decorated Carbon Polyhedrons for Enhanced Microwave Absorption. ACS Appl. Nano Mater..

[22] Wang Y.H., Han X.J., Xu P., Liu D.W., Cui L.R., Zhao H.H., Du Y.C. (2019). Synthesis of Pomegranate-Like Mo_2_C@C Microsphere for Highly Efficient Microwave Absorption. Chem. Eng. J..

[23] Fan X., Liu Y., Peng Z., Zhang Z., Zhou H., Zhang X., Yakobson B.I., Goddard W.A., Guo X., Hauge R.H. (2017). Atomic H-Induced Mo_2_C Hybrid as an Active and Stable Bifunctional Electrocatalyst. ACS Nano.

[24] Huang Y., Gong Q., Song X., Feng K., Nie K., Zhao F., Wang Y., Zeng M., Zhong J., Li Y. (2016). Mo_2_C Nanoparticles Dispersed on Hierarchical Carbon Microflowers for Efficient Electrocatalytic Hydrogen Evolution. ACS Nano.

[25] Zhou P., Chen J.H., Liu M., Jiang P., Li B., Hou X.M. (2017). Microwave Absorption Properties of SiC@SiO_2_@Fe_3_O_4_ Hybrids in the 2–18 GHz Range. Int. J. Min. Met. Mater..

[26] Xu Y., Shen G., Wu H., Liu B., Fang X., Zhang D., Zhu J. (2017). Double-layer Microwave Absorber based on Nanocrystalline CoFe_2_O_4_ and CoFe_2_O_4_/PANI Multi-Core/Shell Composites. Mater. Sci..

[27] Li X.-P., Deng Z., Li Y., Zhang H.-B., Zhao S., Zhang Y., Wu X.-Y., Yu Z.-Z. (2019). Controllable synthesis of hollow microspheres with Fe@Carbon dual-shells for broad bandwidth microwave absorption. Carbon.

[28] An Z., Liu R., Zhang J. (2022). Carbon/Carbon-Ag-Fe_3_O_4_ Dual Shell Hollow Microspheres: High Efficient Pyrolysis Synthesis and Broad Band Microwave Absorption. J. Alloys Compd..

[29] Xu L., Tao J., Zhang X., Yao Z., Zavabeti A., Zhou J. (2022). Co@N-doped Double-Shell Hollow Carbon via Self-Templating-Polymerization Strategy for Microwave Absorption. Carbon.

[30] Wang Y.Q., Zhao H.B., Cheng J.B., Liu B.W., Fu Q., Wang Y.Z. (2022). Hierarchical Ti_3_C_2_T_x_@ZnO Hollow Spheres with Excellent Microwave Absorption Inspired by the Visual Phenomenon of Eyeless Urchins. Nano-Micro Lett..

[31] Wang Y., Yu L., Lou X.W. (2016). Formation of Triple-Shelled Molybdenum-Polydopamine Hollow Spheres and Their Conversion into MoO_2_/Carbon Composite Hollow Spheres for Lithium-Ion Batteries. Angew Chem. Int. Ed. Eng..

[32] Wang Y., Li X., Han X., Xu P., Cui L., Zhao H., Liu D., Wang F., Du Y. (2020). Ternary Mo_2_C/Co/C Composites with Enhanced Electromagnetic Waves Absorption. Chem. Eng. J..

[33] Wang Y.H., Zhang M.H., Deng X.S., Li Z.G., Chen Z.S., Shi J.M., Han X.J., Du Y.C. (2022). Reduced Graphene Oxide Aerogel Decorated with Mo_2_C Nanoparticles Towards Multifunctional Properties of Hydrophobicity, Thermal Insulation, and Microwave Absorption. Int. J. Miner. Metall. Mater..

[34] Gao Q., Zhao X., Xiao Y., Zhao D., Cao M. (2014). A Mild Route to Mesoporous Mo_2_C-C Hybrid Microsphere for High Performance Lithium-ion Batteries. Nanoscale.

[35] Yang L., Li X., Ouyang Y., Gao Q., Ouyang L., Hu R., Liu J., Zhu M. (2016). Hierarchical MoO_2_/Mo_2_C/C Hybrid Nanowires as High-Rate and Long-Life Anodes for Lithium-Ion Batteries. ACS Appl. Mater. Interfaces.

[36] Ge R., Huo J., Sun M., Zhu M., Li Y., Chou S., Li W. (2021). Surface and Interface Engineering: Molybdenum Carbide-Based Nanomaterials for Electrochemical Energy Conversion. Small.

[37] Qutaish H., Tanaka S., Kaneti Y.V., Lin J., Bando Y., Alshehri A.A., Yusa S.I., Yamauchi Y., Hossain M.S.A., Kim J. (2018). Soft-Templated Synthesis of Mesoporous Nickel Oxide using Poly(styrene-block-acrylic acid-block-ethylene glycol) Block Copolymers. Micropor. Mesopor. Mat..

[38] Zhang Z.Y., Cai L.J., Lu W., Chai Y. (2017). Phase and Facet Control of Molybdenum Carbide Nanosheet Observed by In Situ TEM. Small.

[39] Zhang Q., Du Z., Hou M., Ding Z., Huang X., Chen A., Ma Y., Lu S., Tang X.-Z. (2022). Ultralight, Anisotropic, and Self-Supported Graphene/MWCNT Aerogel with High-Performance Microwave Absorption. Carbon.

[40] Du Y., Liu T., Yu B., Gao H., Xu P., Wang J., Wang X., Han X. (2012). The Electromagnetic Properties and Microwave Absorption of Mesoporous Carbon. Mater. Chem. Phys..

[41] Li Y., Liao Y., Ji L., Hu C., Zhang Z., Zhang Z., Zhao R., Rong H., Qin G., Zhang X. (2022). Quinary High-Entropy-Alloy@Graphite Nanocapsules with Tunable Interfacial Impedance Matching for Optimizing Microwave Absorption. Small.

[42] Qiang R., Du Y., Wang Y., Wang N., Tian C., Ma J., Xu P., Han X. (2016). Rational Design of Yolk-Shell C@C Microspheres for the Effective Enhancement in Microwave Absorption. Carbon.

[43] Du Y., Wang J., Cui C., Liu X., Wang X., Han X. (2010). Pure carbon microwave absorbers from anion-exchange resin pyrolysis. Synth. Met..

[44] Bi N., Liu X., Wu N., Cui C., Sun Y. (2015). Improved Electrochemical Performance of Onion-Like Carbon Coated Magnetite Nanocapsules as Electromagnetic Absorptive Anode Materials for Lithium-Ion Batteries. RSC Adv..

[45] Duan W., Li X., Wang Y., Qiang R., Tian C., Wang N., Han X., Du Y. (2018). Surface Functionalization of Carbonyl Iron with Aluminum Phosphate Coating Toward Enhanced Anti-Oxidative Ability and Microwave Absorption Properties. Appl. Surf. Sci..

[46] Zhang X., Ji G., Liu W., Quan B., Liang X., Shang C., Cheng Y., Du Y. (2015). Thermal Conversion of an Fe_3_O_4_@Metal-Organic Framework: A New Method for an Efficient Fe-Co/Nanoporous Carbon Microwave Absorbing Material. Nanoscale.

[47] Lv H., Ji G., Zhang H., Du Y. (2015). Facile Synthesis of a CNT@Fe@SiO_2_ Ternary Composite with Enhanced Microwave Absorption Performance. RSC Adv..

[48] Li X., Yin X., Han M., Song C., Xu H., Hou Z., Zhang L., Cheng L. (2017). Ti_3_C_2_MXenes Modified with in situ Grown Carbon Nanotubes for Enhanced Electromagnetic Wave Absorption Properties. J. Mater. Chem. C.

[49] Yang R., Wang B., Xiang J., Mu C., Zhang C., Wen F., Wang C., Su C., Liu Z. (2017). Fabrication of NiCo-Anchored Graphene Nanosheets by Liquid-Phase Exfoliation for Excellent Microwave Absorbers. ACS Appl. Mater. Interfaces.

[50] Wen F., Zhang F., Liu Z. (2011). Investigation on Microwave Absorption Properties for Multiwalled Carbon Nanotubes/Fe/Co/Ni Nanopowders as Lightweight Absorbers. J. Phys. Chem. C.

[51] Shu R., Wang M., Yang Y., Chang S., Zhang G., Gan Y., Shi J., He J. (2017). Solvothermal Synthesis of Reduced Graphene Oxide/Ferroferric Oxide Hybrid Composites with Enhanced Microwave Absorption Properties. Nano.

[52] Zhang P., Han X., Kang L., Qiang R., Liu W., Du Y. (2013). Synthesis and Characterization of Polyaniline Nanoparticles with Enhanced Microwave Absorption. RSC Adv..

[53] Lv H., Liang X., Ji G., Zhang H., Du Y. (2015). Porous Three-Dimensional Flower-like Co/CoO and Its Excellent Electromagnetic Absorption Properties. ACS Appl. Mater. Interfaces.

[54] Qi X., Hu Q., Cai H., Xie R., Bai Z., Jiang Y., Qin S., Zhong W., Du Y. (2016). Heteronanostructured Co@Carbon Nanotubes-Graphene Ternary Hybrids: Synthesis, Electromagnetic and Excellent Microwave Absorption Properties. Sci. Rep..

[55] Cao Y., Su Q., Che R., Du G., Xu B. (2012). One-Step Chemical Vapor Synthesis of Ni/Graphene Nanocomposites with Excellent Electromagnetic and Eectrocatalytic Properties. Synth. Met..

[56] Jiang Y., Chen Y., Liu Y.-J., Sui G.-X. (2018). Lightweight Spongy Bone-Like Graphene@SiC Aerogel Composites for High-Performance Microwave Absorption. Chem. Eng. J..

[57] Delfini A., Albano M., Vricella A., Santoni F., Rubini G., Pastore R., Marchetti M. (2018). Advanced Radar Absorbing Ceramic-Based Materials for Multifunctional Applications in Space Environment. Materials.

[58] Cheng Y., Li Z., Li Y., Dai S., Ji G., Zhao H., Cao J., Du Y. (2018). Rationally Regulating Complex Dielectric Parameters of Mesoporous Carbon Hollow Spheres to Carry out Efficient Microwave Absorption. Carbon.

[59] Cao M.S., Yang J., Song W.L., Zhang D.Q., Wen B., Jin H.B., Hou Z.L., Yuan J. (2012). Ferroferric Oxide/Multiwalled Carbon Nanotube *vs* Polyaniline/Ferroferric Oxide/Multiwalled Carbon Nanotube Multiheterostructures for Highly Effective Microwave Absorption. ACS Appl. Mater. Interfaces.

[60] Guan X., Yang Z., Zhou M., Yang L., Bagher Aslibeiki R., Ji G. (2022). 2D MXene nanomaterials: Synthesis, mechanism, and multifunctional applications in microwave absorption. Small Struct..

[61] Zhou W., Long L., Xiao P., Li Y., Luo H., Hu W.-D., Yin R.-M. (2017). Silicon Carbide Nano-Fibers in-situ Grown on Carbon Fibers for Enhanced Microwave Absorption Properties. Ceram. Int..

[62] Micheli D., Vricella A., Pastore R., Marchetti M. (2014). Synthesis and Electromagnetic Characterization of Frequency Selective Radar Absorbing Materials Using Carbon Nanopowders. Carbon.

[63] Liu W., Shao Q., Ji G., Liang X., Cheng Y., Quan B., Du Y. (2017). Metal–Organic-Frameworks Derived Porous Carbon-Wrapped Ni Composites with Optimized Impedance Matching as Excellent Lightweight Electromagnetic Wave Absorber. Chem. Eng. J..

[64] Tao J.Q., Zhou J.T., Yao Z.J., Jiao Z.B., Wei B., Tan R.Y., Li Z. (2021). Multi-Shell Hollow Porous Carbon Nanoparticles with Excellent Microwave Absorption Properties. Carbon.

[65] Wang B.L., Wu Q., Fu Y.G., Liu T. (2021). A Review on Carbon/Magnetic Metal Composites for Microwave Absorption. J. Mater. Sci. Technol..

[66] Qiu Y., Yang H., Wen B., Ma L., Lin Y. (2021). Facile Synthesis of Nickel/Carbon Nanotubes Hybrid Derived from Metal Organic Framework as a Lightweight, Strong and Efficient Microwave Absorber. J. Colloid Interface Sci..

[67] Zhang Z., Tan J., Gu W., Zhao H., Zheng J., Zhang B., Ji G. (2020). Cellulose-Chitosan Framework/Polyailine Hybrid Aerogel Toward Thermal Insulation and Microwave Absorbing Application. Chem. Eng. J..

[68] Feng J., Zong Y., Sun Y., Zhang Y., Yang X., Long G., Wang Y., Li X., Zheng X. (2018). Optimization of Porous FeNi/N-GN Composites with Superior Microwave Absorption Performance. Chem. Eng. J..

[69] Micheli D., Pastore R., Apollo C., Marchetti M., Gradoni G., Moglie F., Primiani V.M. Carbon Based Nanomaterial Composites in RAM and Microwave Shielding Applications. Proceedings of the 2009 9th IEEE Conference on Nanotechnology (IEEE-NANO).

[70] Qiang R., Du Y., Chen D., Ma W., Wang Y., Xu P., Ma J., Zhao H., Han X. (2016). Electromagnetic Functionalized Co/C Composites by in situ Pyrolysis of Metal-Organic Frameworks (ZIF-67). J. Alloys Compd..

[71] Meng F., Wei W., Chen X., Xu X., Jiang M., Jun L., Wang Y., Zhou Z. (2016). Design of Porous C@Fe_3_O_4_ Hybrid Nanotubes with Excellent Microwave Absorption. Phys. Chem. Chem. Phys..

[72] Jiang J., Li D., Geng D., An J., He J., Liu W., Zhang Z. (2014). Microwave Absorption Properties of Core Double-Shell FeCo/C/BaTiO_3_ Nanocomposites. Nanoscale.

[73] Liu D., Qiang R., Du Y., Wang Y., Tian C., Han X. (2018). Prussian Blue Analogues Derived Magnetic FeCo Alloy/Carbon Composites with Tunable Chemical Composition and Enhanced Microwave Absorption. J. Colloid Interface Sci..

