# Supplementary material for: Construction of Dual-Shell Mo2C/C Microsphere towards Efficient Electromagnetic Wave Absorption
Source: Int J Mol Sci. 2022 Nov 22;23(23):14502. doi: 10.3390/ijms232314502 (PMC9738143; doi:10.3390/ijms232314502)
Supplement: Supplementary file 1 [file ijms-23-14502-s001.zip › ijms-2019595-SI.pdf]

Supporting Information

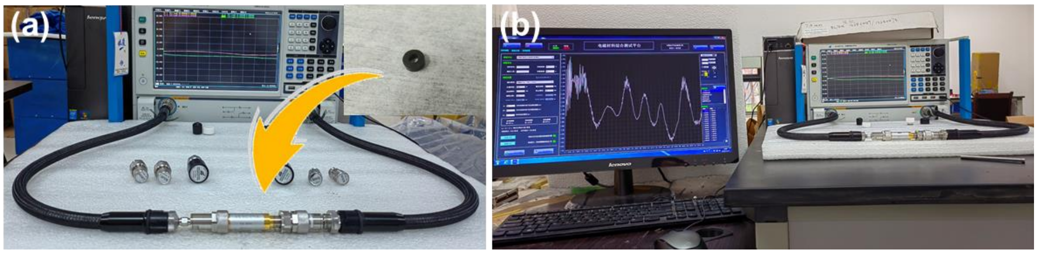

Figure S1. The pictures of coaxial calibration kit and sample (**a**), and measurement procee of Ceyear AV3672C (**b**).

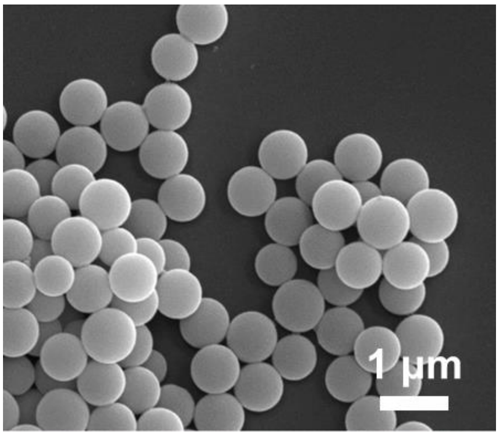

Figure S2. SEM image of Mo-GL nanospheres.

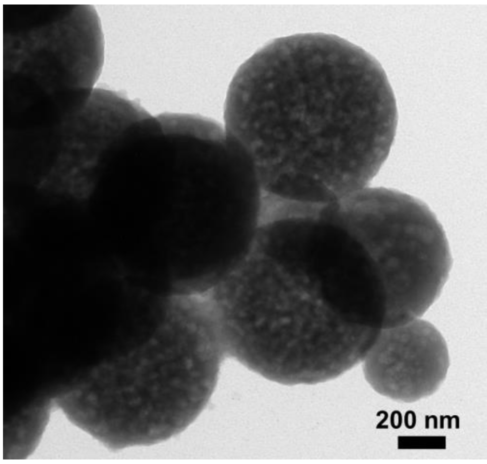

Figure S3. TEM image of Mo-PD nanospheres.

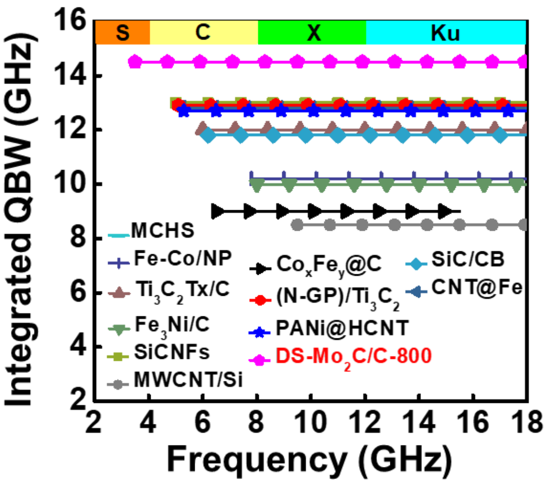

Figure S4. The integrated QBW at the range of 2.0-18.0 GHz of DS-Mo_2C/C-800 and other carbon-based EM absorption materials.

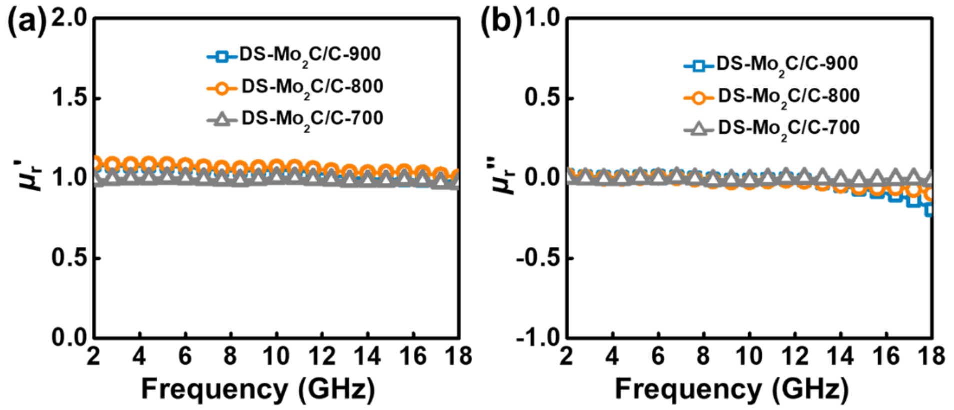

Figure S5. Real parts (**a**) and imaginary parts (**b**) of the complex permeability of DS-Mo_2C/C-700, DS-Mo_2C/C-800, and DS-Mo_2C/C-900.

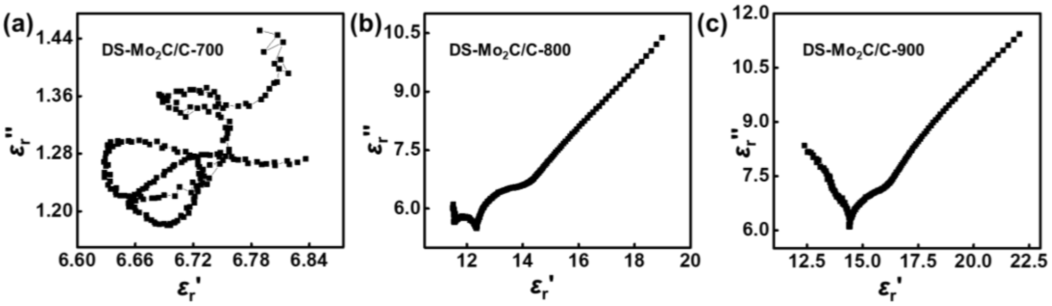

Figure S6. The Cole-Cole semicircle of DS-Mo_2C/C-700 (**a**), DS-Mo_2C/C-800 (**b**), and DS-Mo_2C/C-900 (**c**).
